# 3D-printed model and osteotomy template technique compared with conventional closing-wedge osteotomy in cubitus varus deformity

**DOI:** 10.1038/s41598-022-10732-9

**Published:** 2022-04-26

**Authors:** Jin Li, Jing Wang, Saroj Rai, RenHao Ze, Pan Hong, ShangYu Wang, Xin Tang

**Affiliations:** 1 Department of Orthopaedic Surgery, Union Hospital, Tongji Medical College, Huazhong University of Science and Technology, Wuhan, 430022 China; 2 Department of Radiology, Union Hospital, Tongji Medical College, Huazhong University of Science and Technology, Wuhan, 430022 China; 3 Department of Orthopaedics and Trauma Surgery, Blue Cross Hospital, Tripureswor, Kathmandu, 44600 Nepal

**Keywords:** Outcomes research, Translational research

## Abstract

Cubitus varus deformity is the most common late complication of malunited supracondylar fracture that requires corrective osteotomy and fixation. From 2009 to 2017, 40 consecutive patients with cubitus varus deformity were included. Twenty patients underwent the conventional closing-wedge osteotomy (conventional group), while the other twenty patients underwent the 3D-printed model and osteotomy template osteotomy (3D-printed template group). The functional outcome was evaluated using the Mayo Elbow Performance Index (MEPI) Score and Flynn criteria. There were no statistically significant differences were observed regarding the humerus-elbow-wrist angle and tilting angle between the two groups, both preoperatively and postoperatively at 24 months. No statistically significant differences were observed regarding the elbow ROM (127.0 ± 4.7° VS 128.9 ± 3.8°) and MEPI score (93.5 ± 3.3 VS 94.3 ± 4.1) between the groups. All patients were satisfied both cosmetically and functionally as per the Flynn criteria and MEPI score. The conventional osteotomy and 3D-printed model and osteotomy template techniques both met the treatment requirements of cubitus varus deformity. The 3D-printed template technique showed better osteotomy accuracy, but no significant advantage regarding the functional and cosmetic results than conventional osteotomy.

## Introduction

Cubitus varus deformity is the most common delayed complication of supracondylar fracture of the humerus (SFH)^1–3^. It is caused by malunion of supracondylar fracture, and incidence ranges from 30 to 57%^4–6^. The deformity in cubitus varus is triplanar in nature, which is confirmed by computed tomography (CT) scan where varus in the coronal plane, extension in the sagittal plane, and internal rotation in the axial plane is evident^7,8^. Aesthetic problems are the primary concern for growing children; however, the deformity might be associated with impaired function and even result in joint instability^9,10^, loss of elbow flexion and tardy ulnar nerve palsy^11–16^.

Several surgical techniques for corrective osteotomy of cubitus varus deformity of the humerus have been reported. Such osteotomy techniques include medial open wedge osteotomy^17^, lateral closing wedge osteotomy^18,19^, reverse V osteotomy^20,21^, dome osteotomy^22^, coronal and sagittal plane-based closing wedge osteotomy^23,24^, These techniques are technically simple and widely performed, but only correct the varus or varus and extension components of the deformity. Initially, it was thought that the correction of internal rotation is not necessary^25^. However, there have always been potential risks of complications^26–30^ such as tardy ulnar nerve palsy^12–14^ and abnormal elbow motion. Such complications might have resulted from the rotational deformity^26^ and the rotational deformity can be successfully addressed with a computer-assisted 3-dimensional printed (3D-printed) osteotomy guide plate^26–28^. However, preoperative planning and accurate surgical techniques are always challenging.

The 3D computer simulation based on CT scan or magnetic resonance imaging (MRI) data have been demonstrated previously^31–33^. The 3D-printed model and osteotomy template based on computer simulation for the corrective osteotomy is a recent advancement and has been recommended as an ideal choice for cubitus varus deformity^32–35^. However, no previous study has directly compared the result of conventional osteotomy with the 3D-printed model and template guide osteotomy using the same surgical technique.

This study aimed to compare the accuracy of deformity correction and functional outcomes of patients with cubitus varus deformity undergoing the conventional osteotomy and 3D-printed model and template guide osteotomy.

## Materials and methods

### Patients

From July 2009 to December 2017, 40 consecutive patients, including 32 males and 8 females were retrospectively reviewed. All of these patients had a previous history of SFH, resulting in cubitus varus deformity. Among them, 15 patients were managed with closed reduction and percutaneous pinning (CRPP) with Kirschner wire (K-wire), and rest were managed conservatively. The patients were divided into two groups, (1) conventional osteotomy (conventional group) and (2) 3D-printed model and template guide osteotomy (3D-printed template group), as per the surgery they underwent. Out of 40 patients, 20 patients were included in the conventional group, and the remaining 20 patients were included in the 3D-printed template group.

All the patients who underwent corrective osteotomy using either of these techniques and followed up for at least 24 months were included in the study. Patients with any associated injuries with the initial fractures such as neurovascular injury were not included. The preoperative data were collected from the hospital database, and the postoperative data were collected during the follow-up visit. To avoid the susceptibility of bias, a Ph.D. scholar who was not involved in the surgery performed all the clinical evaluations. The study was approved by the Ethics Committee of Tongji Medical College, Huazhong University of Science and Technology (IORG No: IORG0003571). All methods were performed in accordance with the relevant guidelines and regulations. Written informed consent was obtained from the patients' legal guardians after they were informed about the purpose and procedure of the study.

### 3D reconstruction and osteotomy simulation

Standard anteroposterior (AP) and lateral view radiograph and CT scan of ipsilateral and contralateral upper limbs in full extension and supination were taken in all patients preoperatively. The CT images were reconstructed with a dual-source helical-type CT system (Siemens, Germany) with a tube voltage of 120 kV and scan pitch 0.625 mm as scanning parameters. Bilateral humerus-elbow-wrist angle and tilting angle were measured on plain radiographs. The humerus-elbow-wrist angle is defined as a line passing through midpoints of the radius and ulna from proximal to distal and the humerus longitudinal axis on AP radiographs. The tilting angle is defined as the long humerus axis with respect to the articular condyles anterior tilt on the lateral radiographs^7,32,36^.

**Figure 1:**
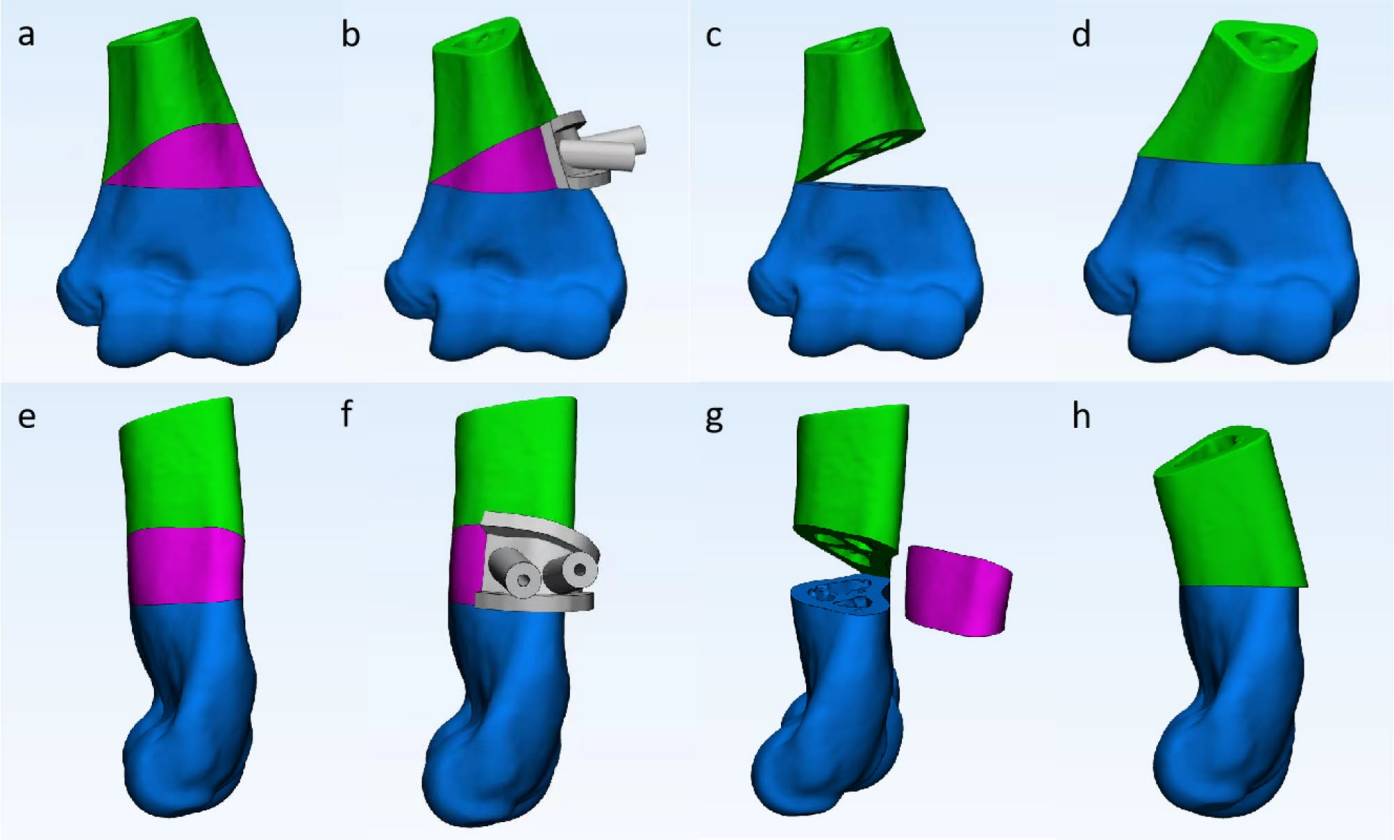
Osteotomy simulation with 3D printing template and the process of correction. The distal osteotomy plane was parallel to the elbow joint line above the olecranon fossa, and the proximal osteotomy plane was determined by the calculated osteotomy angle previously. Antero-posterior (**a**) and lateral view (**e**) of the wedge-shaped segment was formed by the two planes intersected at the medial bone cortex (purple area). Antero-posterior (**b**) and lateral view (**f**) of osteotomy template design according to the wedge-shaped segment and its removal [(**c)** and (**g**)]. Antero-posterior (**d**) and lateral view (**h**) after reduction of the distal segment of the humerus.

Digital CT image data of bilateral upper limb in 3D-printed template osteotomy group were copied to a workstation (Lenovo Blade 7000; Lenovo, Huizhou, Guangdong) in Medicine (DICOM) format and input into the Mimics18.0 software (Materialize, Leuven, Belgium) to obtained three-dimensional reconstructed models of the humerus, radius, and ulna. The angle and width of wedge osteotomy were calculated as the measurement on the contralateral upper limb, which equaled the degrees of ulno-humeral varus plus the degree of the humerus-elbow-wrist angle of the contralateral upper limb on the coronal plane, and the difference value of tilting angle on the sagittal plane. The process of correction was simulated as follows (Fig. 1a through h). The distal osteotomy plane was parallel to the elbow joint line above the olecranon fossa, and the proximal osteotomy plane was determined by the previously calculated osteotomy angle. A wedge-shaped segment was formed by the two planes intersected at the medial bone cortex. The simulated osteotomy was completed by removing the wedge-shaped segment and reducing the distal fragment of the humerus on the proximal part. The distal fragment could be translated medially and de-rotated accordingly to address the rotational deformity and minimize the lateral condyle prominence. The humerus-elbow-wrist angle, tilting angle, and residual rotational deformity were measured again after simulated osteotomy to ensure the adequate correction of the cubitus varus deformity. The residual rotational deformity should be less than 15 degrees^32^.

### 3D model and osteotomy template design and production

**Figure 2:**
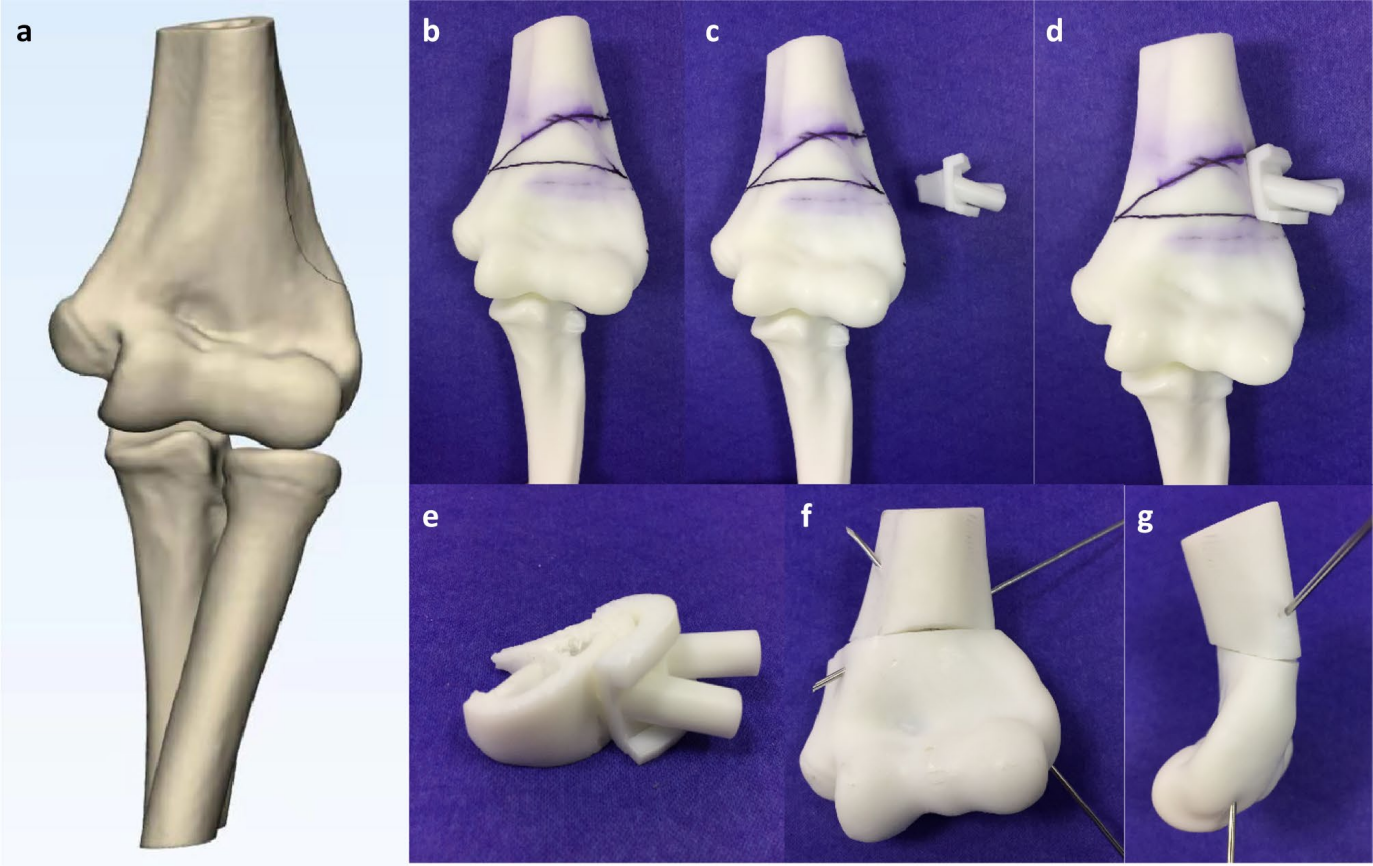
Three-dimensional reconstructed models of the humerus, radius, and ulna (**a**). Simulation of the process of correction preoperatively: the 3D printing model of humerus, marking osteotomy line and with 3D printing template [(**b)**, (**c**) and (**d**)]; the osteotomy template removed with an osteotomized bone wedge (**e**); anteroposterior (**f**) and lateral view (**g**) after reduction of the distal segment of the humerus.

The CT image data of the bony surface of the distal humerus were extracted and formulated a 3D simulation model with authorized software (Magics RP; Materialise, Leuven, Belgium). The osteotomy template was established as per the shape of the distal humerus simulation model, and two k-wire guide holes were imported into the osteotomy template. It was designed in such a way that a wedge-shaped segment along with the guide plate can be removed after the completion of the osteotomy (Fig. 1). The designed 3D simulation model and osteotomy template data were saved in STL format and printed with materials of photosensitive resin by the 3D printer (Lite 450, KangDeYou Med, Inc., Wuhan, Hubei, China) (Fig. 2 a). The 3D-printed model and osteotomy template could be used for simulation of the process of correction preoperatively. (Fig. 2b through g).

### Surgical technique

An experienced surgeon performed all the surgeries. All patient's guardians were clearly explained about the pros and cons of both the surgical techniques and let them choose whether to make a 3D-printed model and osteotomy template or not. Under general anaesthesia, the patient was positioned in supine. A tourniquet was routinely applied and inflated at 200 mmHg. A limited lateral incision, extending from 4 cm proximal to 2 cm distal to the elbow crease, was made. Radial nerve injury was avoided by adequate exposure of the soft tissue (3 cm) above and below the osteotomy site but not to identify the radial nerve.

In the conventional group, two k-wires (2 mm) were placed above and below the desired osteotomy line, which was confirmed by intraoperative fluoroscopy. The distal osteotomy was made just above the olecranon fossa parallel to the elbow joint line. Whereas, the proximal osteotomy was made as per the preplanned angle on preoperative AP and lateral radiographs. After osteotomy, a wedge-shaped bone was removed, and both the k-wires were brought parallel to each other to assure adequate reduction. If the osteotomy angle was more than 30 degrees, the ulnar nerve transposition was performed to avoid iatrogenic injury.

In the 3D-printed template group, the proximal and distal osteotomy sites were determined by the 3D-printed osteotomy template held correctly onto bone by two k-wires (1.5 mm or 2 mm). The osteotomy was performed along the superior and inferior surface of the template so that the k-wires and template were removed along with the wedge-shaped osteotomized segment. Another two lateral k-wires (1.5 mm or 2 mm) were inserted in proximal and distal fragments close to the wedge-shaped osteotomized segment. These two k-wires were inserted in such a way that upon removal of the osteotomized segment these can be rotated and brought parallel together so that it can correct the deformity in both the coronal and axial plane.

Once the satisfactory osteotomy angle was achieved in both the groups, the fragments were internally fixed with a 4-hole 3.5 mm locking plate (Trauson, ChangZhou, China). Bicortical purchase was made for proximal screws, but distal screws were used only unicortical to avoid ulnar nerve injury in the far cortex. The stability of the fixation was confirmed under fluoroscopy in maximum flexion and extension. Final check AP and lateral radiographs were obtained before the wound closure.

### Postoperative care and follow-up

A similar rehabilitation program was applied in both groups. An above elbow back slab was applied for 1–3 weeks, until the swelling and pain subsided, with a forearm at 90 degrees of flexion and mid-prone position. Rehabilitation was started with a gentle elbow range of motion (ROM) exercise at home as per the surgeon’s instructions. Patients were followed up at 6-week, 3-month, 6-month, 1-year, and yearly after that. At each follow-up visit, standard AP and lateral radiographs were taken along with the clinical evaluation postoperatively. The plate was removed after the clinical and radiological evidence of union and was usually 6 to 12 months postoperatively.

The radiological union is defined by the absence of the osteotomy gap in at least three out of four cortices^37^. Delayed union is defined by the presence of a visible osteotomy gap in more than one of four cortices on AP and lateral radiographs at 12 weeks^38^. Patients were clinically and radiologically evaluated at the last follow-up visit. Clinical evaluations included elbow ROM, elbow-carrying angle, and complications, including neurovascular and surgery-related complications. Functional assessment was evaluated by the Mayo Elbow Performance Index (MEPI) score^39^ and Flynn criteria^40^. We considered the excellent to fair result as satisfactory. The additional cost for the 3D-printed template group was evaluated in USD.

### Statistical analysis

The data of conventional and 3D-printed template groups were compared and statistically analyzed using the method of Mann–Whitney U test for numerical variables and Fisher's exact test for categorical variables with SPSS (version 22.0; SPSS, Chicago, IL, USA). The level of significance was set at a p-value < 0.05.

## Results

Table 1 shows the demographic and clinical details of the patients. There was no statistically significant difference observed regarding the age, gender, time to union, and follow-up period between the groups (*p* > 0.05). The average operation duration was significantly shorter (53 min) in the conventional group than the 3D-printed template group (60 min) (*p* < 0.001). No statistically significant differences were observed regarding the humerus-elbow-wrist angle and tilting angle between the two groups, both preoperatively and postoperatively at 24 months. However, the humerus-elbow-wrist angle and tilting angle showed a statistically significant difference between the two groups when compared with the contralateral side at 24 months postoperatively (*p* = 0.002 and *p* < 0.001, respectively). No statistically significant differences were observed regarding the elbow ROM and MEPI score between the groups. All patients were satisfied both cosmetically and functionally as per the Flynn criteria and MEPI score (Table 2). However, both groups did not show any statistically significant differences at the last follow-up. The 3D-printed template group had an additional cost of USD.500.

**Table 1:** Comparisons between conventional and 3D printing template groups.

Variables	Groups		
	Conventional	3D Template	p*
Number of patients	20	20	–
Age (year)	9.3 ± 6.2	8.9 ± 3.3	0.714
Male	14 (70%)	18 (90%)	0.235
Operation duration (min)	50.6 ± 6.4	60.4 ± 5.4	< 0.001
Time to union (week)	10.6 ± 1.7	10.7 ± 1.6	0.868
Follow-up (month)	36.9 ± 11.9	37.2 ± 10.7	0.978
**Humerus-elbow-wrist angle (°)**			
Pre-op	− 20.4 ± 7.3	− 19.1 ± 6.1	0.522
24th month follow up	11.4 ± 3.5	10.6 ± 4.5	0.667
Difference with unfectted side	3.4 ± 1.5	2.0 ± 1.5	0.002
**Tilting angle (°)**			
Pre-op	33.6 ± 10.9	33.6 ± 11.2	0.776
24th month follow up	38.6 ± 3.6	38.4 ± 3.8	0.877
Difference with unfectted side	9.8 ± 2.1	5.7 ± 3.0	< 0.001
**Arc of motion (°)**			
Pre-op	126.0 ± 8.0	127.0 ± 7.8	0.728
Post-op	127.0 ± 4.7	128.9 ± 3.8	0.175
MEPS score	93.5 ± 3.3	94.3 ± 4.1	0.442

*Mann–Whitney U test and Fisher’s exact test.

**Table 2:** Distribution of cases according to criteria of Flynn and MEPS in two groups.

	Flynn functional *N* (%)		Flynn cosmetic *N* (%)		MEPS *N* (%)	
	Conventional	3D	Conventional	3D	Conventional	3D
**Satisfactory**						
Excellent (0–5°/ > 90)	(10/50)	(11/55)	(17/85)	(18/90)	(20/100)	(19/95)
Good (6–11°/75–89)	(10/50)	(9/45)	(3/15)	(2/10)	(0)	(1/5)
Fair (11–15°/60–74)	(0)	(0)	(0)	(0)	(0)	(0)
**Unsatisfactory**						
Poor	> 15°(0)	(0)	> 15°(0)	(0)	< 60 (0)	< 60 (0)
P*	1.00		1.00		1.00	

*Fisher's exact test.

No surgery-related complications such as neurovascular injury, myositis ossificans, non-union, recurrence, and other complications requiring further revision were identified during the follow-up visit. There was no noticeable difference present in muscle strength when compared with the uninjured limb clinically at the final follow-up.

## Discussion

To the best of our knowledge, this is the first-ever study to compare the results of conventional osteotomy with the 3D-printed template osteotomy guide in patients with cubitus varus deformity^35^. The SFH is the most commonly operated fracture during childhood, which counts for 17.9% of all pediatric fractures as reported^2,41^. The most common late complication of SFH is the cubitus varus deformity that results from the malunion of the fractures. Various osteotomy techniques have been described in the literature and can be divided into four parts, including lateral closing wedge osteotomy^6,18,19^, dome osteotomy^22,42^, complex (multiplanar) osteotomy^9,17,26^, and distraction osteogenesis^38,43^. The complications following the corrective osteotomy range from 13 to 34%, and increase with the complexity of the surgical technique^43^. No technique has been reported to be significantly better or safer than others^44^.

However, conventional osteotomy techniques always have shortcomings of difficult preoperative planning and inadequate deformity correction. To overcome these shortcomings, computer simulation with CT scan or MRI data have been recommended for accurate preoperatively planing^29,31–33^. Recently, several articles have been published based on this technology, using the 3D-printed model and the osteotomy template^8,32–35^. The authors reported excellent outcomes with improved accuracy in the osteotomy, so they recommended this technique in cubitus varus deformity as an ideal treatment choice. However, there has not been any published literature, so far, comparing outcomes of conventional and 3D-printed template techniques. Despite the similar demographic characteristics of our patients, the 3D-printed template group did not show any better results in terms of humerus-elbow-wrist angle and tilting angle at the last follow-up.

The conventional osteotomy is planned as per the measurement of the varus deformity angle on plain radiographs. CT images may help the surgeon to plan for optimum osteotomy site in a 3D manner. The correction of deformity following osteotomy relies on the operating surgeon's experience. Deformity correction should be in the form of changing the varus into valgus, extension into flexion, and internal rotation into external rotation.

The 3D-printed template technique has the theoretical advantage of a relatively accurate and easier osteotomy. The 3D-printed osteotomy guide plate was fixed onto the desired osteotomy site of the distal humerus, followed by osteotomy. However, there has always been difficulty performing osteotomy as no specialized saw can quickly complete the osteotomy by avoiding the template. This technique might provide a relatively better osteotomy angle^35^, and possibility to achieve the same angle as in the contralateral site. However, even this technique utilizes the same principle as in the conventional group to correct the deformity in 3-planes. Because of such difficulty, the 3D-printed template group had a significantly longer surgical duration than the conventional group. This result was inconsistent with the report by Zhang et al.^35^ of the 3D-printed template group had a shorter operating time. This problem may be resolved by the improvisation of equipment such as osteotomy slits and the use of a long saw.

Because of the reasons mentioned above, our study provides evidence that the 3D-printed model and osteotomy template has no more added advantage as compared with the conventional osteotomy regarding the accuracy of the corrective osteotomy for an experienced surgeon. Another disadvantage of the 3D-printing model and osteotomy template is the requirement of additional equipment and software for osteotomy simulation and 3D printing of the model and osteotomy template. This technology might not be available in economically underdeveloped areas. The additional cost of the preparation and implementation of the 3D templating system is another issue because it appears not to be justified.

In our study, both the conventional and 3D-printed template groups displayed safe osteotomy techniques for the treatment of cubitus varus deformity. Regardless of osteotomy techniques, satisfactory functional and cosmetic results could be achieved in both groups.

The 3D-printed model and the osteotomy template is a result of technological advancement that might have better accuracy, shorter learning curves, and easier for young surgeons. However, it is more expensive, and this technology may not be available in many countries, especially in developing countries. Even if this technology is available, medical insurance may not cover the extra cost. In contrast, conventional osteotomy has the advantage of readily available surgical procedures and is economical for needy patients.

Although it is the first-ever study to compare the conventional corrective osteotomy vs. 3D-printed osteotomy guide plate, several shortcomings exist. It is a retrospective study with a limited number of patients and a relatively short follow-up period. There might be subjective bias for being a non-randomized study, and the surgical techniques were chosen by the patient's legal guardians.

## Conclusion

The conventional osteotomy and 3D-printing of model and osteotomy template techniques both met the treatment requirements of cubitus varus deformity. The 3D-printing template technique showed better osteotomy accuracy, but no significant advantage regarding the functional and cosmetic results than conventional osteotomy when the surgeon is experienced one. Although this technique brings the additional cost, the preparation and implementation of the 3D templating system are most useful when the surgeon is inexperienced.

## Data Availability

The datasets generated during and/or analyzed during the current study are available from the corresponding author on reasonable request.
